# An Urchin-Shaped Copper-Based Metalloporphyrin Nanosystem as a Sonosensitizer for Sonodynamic Therapy

**DOI:** 10.3390/nano12020209

**Published:** 2022-01-10

**Authors:** Aiqing Ma, Hui Ran, Jiaxing Wang, Rui Ding, Chengyu Lu, Lanlan Liu, Yingmei Luo, Huaqing Chen, Ting Yin

**Affiliations:** 1 Guangdong Provincial Key Laboratory of Research and Development of Natural Drugs, School of Pharmacy, Guangdong Medical University, Dongguan 523808, China; aqma@gdmu.edu.cn (A.M.); hui.ran@siat.ac.cn (H.R.); jiaxingwang1998@163.com (J.W.); dingrui184@163.com (R.D.); luchengyu@gdmu.edu.cn (C.L.); 13650439125@163.com (Y.L.); 2 Guangdong Key Laboratory of Nanomedicine, CAS-HK Joint Lab of Biomaterials, Shenzhen Engineering Laboratory of Nanomedicine and Nanoformulations, Institute of Biomedicine and Biotechnology, Shenzhen Institute of Advanced Technology (SIAT), Chinese Academy of Sciences, Shenzhen 518055, China; ll.liu@siat.ac.cn; 3 Shenzhen Key Laboratory of Gene and Antibody Therapy, Center for Biotechnology and Biomedicine, State Key Laboratory of Chemical Oncogenomics, State Key Laboratory of Health Sciences and Technology, Institute of Biopharmaceutical and Health Engineering, Shenzhen International Graduate School, Tsinghua University, Shenzhen 518055, China

**Keywords:** sonodynamic therapy, copper-based metalloporphyrin, sonosensitizer, urchin-shaped, noninvasive therapy

## Abstract

Sonodynamic therapy (SDT), as a novel cancer therapy strategy, might be a promising approach due to the depth-penetration property in tissue. Sonosensitizers are the key element for efficient SDT. However, the development of sonosensitizers with strong sonosensitization efficacy is still a significant challenge. Herein, an urchin-shaped copper-based metalloporphyrin liposome nanosystem (FA–L–CuPP) is constructed and identified as an excellent sonosensitizer. Under ultrasound (US) irradiation, FA–L–CuPP can be highly excited to generate several reactive oxygen species (ROS), such as singlet oxygen (^1^O_2_) and free radicals (⋅OH). The molecular orbital distribution calculations reveal that a strong intramolecular charge transfer might occur in the CuPP complex under US irradiation, which could afford enough energy to the surrounding O_2_ and H_2_O to concert ^1^O_2_, O_2_^−^ and ⋅OH. Working as “ammunitions”, the largely produced ROS can kill 4T1 tumor cells, effectively inhibiting tumor growth. This work provides an urchin-shaped nanosonosensitizer based on a copper complex, which might provide an idea to design a novel sonosensitizer for noninvasive and precise SDT antitumor applications.

## 1. Introduction

Due to its high morbidity and lethality, cancer is still a significant health issue worldwide [1]. Traditional clinical modalities for cancer treatment such as radiotherapy, chemotherapy and surgery are radioactive, non-specific or invasive, which have severe adverse effects [2]. Therefore, the development of novel therapeutic modalities with high efficiency and mitigated side effects is very significant in anticancer treatments [3,4,5,6].

Photodynamic therapy (PDT) has garnered great attention because of the apparent advantages of the minimally invasive, site-specific activation and easy operation [7,8,9]. The effectiveness of PDT depends on cytotoxic reactive oxygen species (ROS) generated from photosensitizers (PSs) under light irradiation [10,11]. However, due to the weak penetration ability of light, the efficacy of PDT is hindered since the PSs cannot be effectively excited to generate ROS in deep tissue. This obstacle was resolved by sonodynamic therapy (SDT), where ultrasound (US) irradiation is used to overcome the limited tissue penetration ability of light in PDT [12,13,14]. As SDT has benefited from the US-mediated deep tissue penetration, it has become a promising strategy in deep-seated tumor treatment [15,16].

As a development based on PDT, SDT has a similar therapeutic mechanism by exciting sonosensitizers to generate ROS under US treatment to kill tumor cells [17,18]. Therefore, successful SDT relies on the performance of sonosensitizers [19,20]. As reported in the literature, the first-generated and most commonly used sonosensitizers are porphyrins and their derivatives such as protoporphyrin IX (PpIX) and chlorin e6 (Ce6), which generally originate from PSs [13,21,22]. Porphyrins have many unique properties (such as catalytic performance, broad-ranging optoelectronic and large electron conjugated systems, etc.) that have made them be extensively studied in SDT [23]. Recent studies revealed that the features of porphyrins could be readily modulated by various metal coordination inside the porphyrin ring [24,25]. For instance, manganese-coordinated porphyrin complexes exhibited a good SDT effect, and realized the real-time monitoring of tumor accumulation and targeting for precision theranostic SDT [26,27]. Despite rapid development, SDT is still not used in clinical practice, mainly because of its limited therapeutic effect and dubious mechanism. Moreover, the shortcomings of small molecule sonosensitizers (such as instability and low bioavailability, poor biocompatibility, fast elimination from the body) have resulted in reluctant SDT efficiency and hindered their further application. Therefore, developing novel sonosensitizers and modifying small molecule agents to enhance the efficient accumulation in tumor regions would be significant in SDT [28,29].

**Figure 1:**
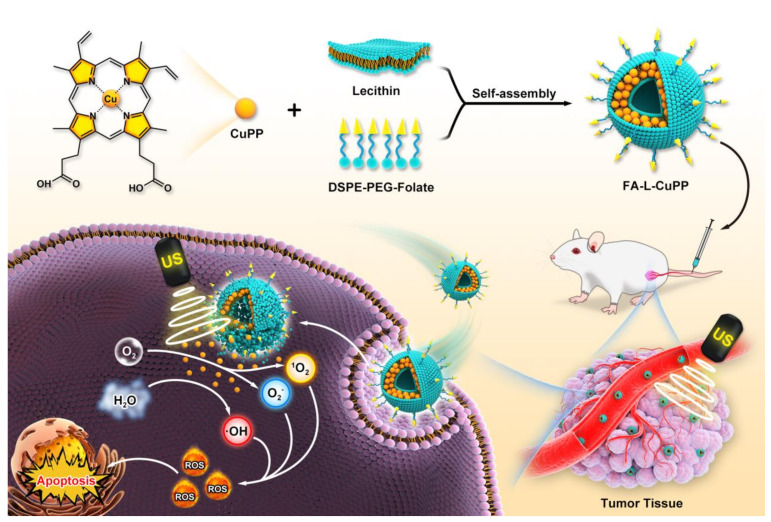
Schematic description of FA–L–CuPP-mediated SDT against tumors. The FA–L–CuPP nanoparticles were first prepared by encapsulating CuPP complex in folate-liposomes. Benefiting from the folate-mediated tumor target recognition, FA–L–CuPP nanoparticles were highly accumulated in tumor at first, and then CuPP complex was activated by US irradiation, which might release enough energy to induce surrounding small molecules to generate amounts of ROS (such as ^1^O_2_, O_2_^−^ and ⋅OH), suppressing the tumor’s growth.

Accordingly, we reported a sonosensitizer based on the metalloprotoporphyrin complex, in which copper ion was coordinated in protoporphyrin IX ring (CuPP). To overcome the insolubility and poor biocompatibility, liposome, as a preferred carrier for encapsulating hydrophilic or lipophilic drugs in the matrix, was used to load the CuPP complex to improve the biostability and minimize the damage to surrounding normal tissues, obtaining nanoparticles of liposome-CuPP (L-CuPP). To improve the target ability to tumor cells, folate (generally overexpressed in most tumors) was inserted into the phopholipid layer of liposomes, constructing a copper-based metalloporphyrin nanosonosensitizer system (FA–L–CuPP) (Figure 1). Experiments showed that FA–L–CuPP could be excited under US irradiation, and ROS generation was validated by electron spin resonance (ESR). Obvious ^1^O_2_ and free radical generation suggested good SDT efficiency. The molecular orbital distribution calculations indicated both the HOMO and LUMO orbitals of CuPP complex were well delocalized among the whole porphyrin rings, while LUMO orbital was more delocalized to the metal center. The amplified delocalization indicated strong intramolecular charge transfer in the CuPP complex. In vitro and in vivo experiments identified that FA–L–CuPP had a good accumulation and efficient killing of the tumor cells under US treatment. Therefore, this work explores the SDT efficiency of a copper-based complex with liposome carriers. This study provides an idea for developing novel sonosensitizers and enriching the SDT mechanism.

## 2. Results and Discussion

### 2.1. Preparation and Characterization of FA–L–CuPP Nanoparticles

**Figure 2:**
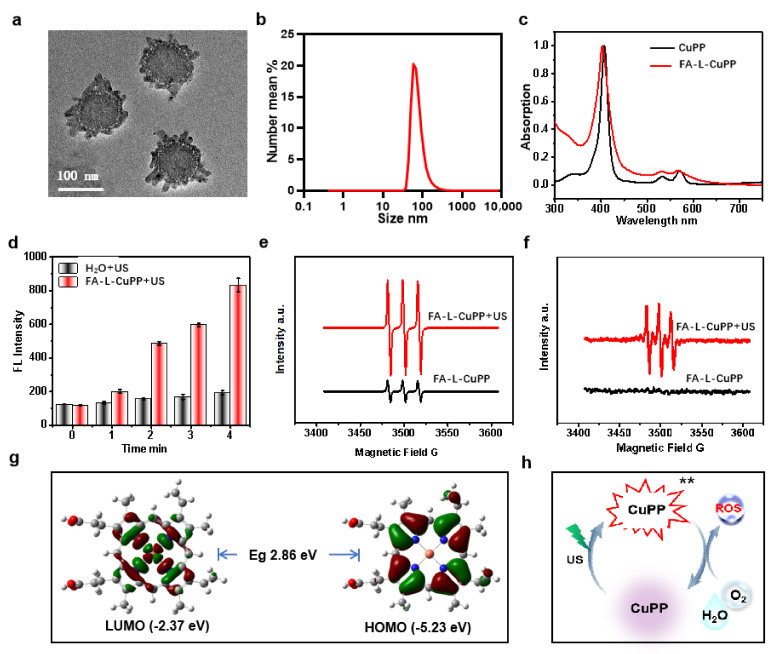
Characterization and US-responsive behavior of FA–L–CuPP under US treatment (1.0 W·cm^−2^, 50% duty cycle): (**a**) TEM images of FA–L–CuPP, showing urchin−shaped structures. (**b**) The size distribution of the FA–L–CuPP determined by DLS. (**c**) UV−Vis spectra of the nanoparticles of FA–L–CuPP, showing the same S− and Q−bands absorption from CuPP complex. (**d**) The FL signal intensity to detect the time−dependent ^1^O_2_ generation by US treatment (the concentration of CuPP complex: 40 μg·mL^−1^). (**e**) ESR spectra to detect ^1^O_2_ generation after US treatment using TEMPO as a probe, and ^1^O_2_ was significantly observed under US irradiation. (**f**) ESR spectra to detect ⋅OH radical group after US irradiation using DMPO as a probe. (**g**) HOMO/LUMO orbitals of CuPP complex calculated by density functional theory (DFT) at the B3LYP Hybrid function, the 6-311G* (C, N, H, O) and def2tzvp (Cu) basis level. (**h**) Diagram illustration for the possible ROS generation mechanism under US treatment.

The preparation strategy for FA–L–CuPP was followed to combine thin-film hydration with sonication. Typically, a liposome mixture containing lecithin and cholesterol was dissolved in chloroform (CHCl_3_). Under stirring, CuPP methanol solution was added to the above mixture. After drying the solution under rotary evaporation, a lipid L-CuPP film was prepared. The FA–L–CuPP film was obtained with a similar method with DSPE-PEG2000-folate solution added. The film was dispersed in ultrapure water again, and the FA–L–CuPP nanoparticles were obtained by sonication. The morphology was first explored by transmission electron microscopy (TEM) imaging. Figure 2a shows that FA–L–CuPP exhibited urchin-shaped feature with good monodispersity. Considering the preparation process, the osmosis of NaHCO_3_ solution during pH adjustment might be responsible for the shape. The average hydrodynamic diameter was also performed to evaluate the size of the nanoparticles, which was around 95 nm (Figure 2b). The appropriate size of FA–L–CuPP would be beneficial for targeting and penetrating tumor tissues through the enhanced permeability and retention (EPR) effect [30,31]. The UV-Vis spectrum of FA–L–CuPP was then explored. Figure 2c reveals that FA–L–CuPP had a similar absorption spectra with the CuPP complex with the S-band absorption at about 410 nm and two Q–bands absorption at about 540 nm and 570 nm, demonstrating good encapsulation of the complex of CuPP. The encapsulation efficiency is 93.4 ± 4.6%, detected by UV–Vis spectrum. The high encapsulation efficiency is beneficial for enhancing the bioavailability of the CuPP complex.

### 2.2. In Vitro SDT Efficiency FA–L–CuPP Nanoparticles

Metalloporphyrins have been reported to exhibit good SDT efficiency. Here, we assumed that FA–L–CuPP also could be excited by US treatment. Singlet oxygen (^1^O_2_) generation was first detected using SOSG as a probe under US treatment to evaluate the US-responsive behavior of FA–L–CuPP. Figure 2d shows that US irradiation induced ^1^O_2_ generation, and the fluorescence (FL) intensity increased over time, indicating a good US response. Electron spin resonance (ESR) spectrum (TEMPO as a ^1^O_2_ probe) was also conducted after the exposure to US treatment to evaluate the ^1^O_2_ generation ability of FA–L–CuPP further. The ESR signal would be split into three narrow lines after interaction between the unpaired electronic spin and the nitrogen ^14^N nucleus in TEMPO [32]. As shown in Figure 2e, characteristic ^1^O_2_-induced signals were significantly observed in the FA–L–CuPP+US group. Notably, without US irradiation, FA–L–CuPP also had weak signals, probably due to the exposure to light during the tests, since porphyrins are also photosensitizers. Moreover, the ⋅OH free radical was also detected under US exposure using 5,5-dimethyl-1-pyrroline N-oxide (DMPO) as a ⋅OH probe. From Figure 2f, an obvious signal was observed after US irradiation, indicating the generation of ⋅OH free radical. However, when compared with the spectrum of ^1^O_2_ detection, the signal peak of ⋅OH free radical was weak. The molecular orbital distribution was then calculated by density functional theory (DFT). In Figure 2g, both the HOMO and LUMO orbitals of CuPP complex are well delocalized among the whole porphyrin rings, while LUMO orbital is more delocalized onto the metal center. The amplified delocalization indicated strong intramolecular charge transfer within the CuPP complex, which might be easily excited under energy. Porphyrin derivates have been well known as effective photosensitizers to induce ^1^O_2_ generation under light energy [33,34]. A similar possible mechanism might be explained in that US excitation energy irradiated metalloporphyrin to a triplet excited state, which in turn was intercepted effectively by the surrounding small molecules (such as oxygen and H_2_O) to generate ROS for SDT antitumor (Figure 2h) [35,36].

**Figure 3:**
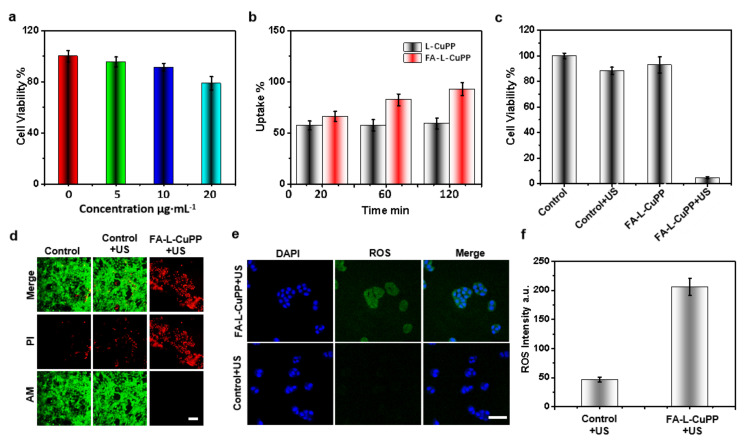
In vitro SDT effect of the FA–L–CuPP against 4T1 cells: (**a**) the in vitro cytotoxicity of the FA–L–CuPP with different concentrations against 4T1 cells. (**b**) The uptake of the FA–L–CuPP to the 4T1 cells (CuPP concentration of 10 µg⋅mL^−1^). (**c**) The sonocytotoxicity of the FA–L–CuPP (10 µg⋅mL^−1^) against 4T1 cells under US treatment (1.0 W·cm^−2^, 50% duty cycle, 2 min). (**d**) The FL imaging of 4T1 cells stained by PI and calcein−AM after various treatments; dead/later apoptosis cells were stained with PI (red), and viable cells were stained with calcein−AM (green) (scale bar = 25 µm). (**e**) ROS imaging in 4T1 cells (DCFH−DA as a probe) after different treatments detected by CLSM (scale bar = 50 µm). (**f**) The intracellular quantitation of ROS generation after different treatments.

The US-excited response of FA–L–CuPP was also carried out through the SDT effect on antitumor cells. Initially, the biosafety of FA–L–CuPP on 4T1 cells was evaluated by a CCK8 assay. Figure 3a shows that FA–L–CuPP induced negligible toxicity below the concentration of 10 μg⋅mL^−1^. Using this concentration, the target ability of FA–L–CuPP was explored by flow cytometric analysis. As shown in Figure 3b, when compared with the nanoparticles of L−CuPP without folate, FA–L–CuPP showed significant target uptake over time, and the uptake quantity was higher than L−CuPP, suggesting good target ability. Subsequently, FA–L–CuPP−induced sonotoxicity was determined. From the data in Figure 3c, 4T1 cells were biosafe without US treatment, and cells were not damaged with simple US irradiation. However, 92% of the killing of the cells occurred in FA–L–CuPP+US group. The significant damage suggests the excellent SDT effect of FA–L–CuPP.

The sonotoxicity of FA–L–CuPP against 4T1 cells was characterized using FL microscopy imaging (Figure 3d). In the control group and the control+US group, negligible red FL signal (dead cells) and a strong green FL signal (live cells) proved that the cells were in an intact physiological state. In contrast, FA–L–CuPP-mediated SDT caused dead cells in the FA–L–CuPP+US group. Good ^1^O_2_ generation in solution under US irradiation reminded us to explore the mechanism of killing 4T1 cells by evaluating the intracellular ROS levels using DCFH−DA. Figure 3e shows that, compared with the simple US treatment group, the FA–L–CuPP+US group presented a strong green FL signal in cells, suggesting an abundance of ROS generation. As an active species, ROS is responsible for cell killing. The ROS generation was also analyzed by flow cytometric analysis. From Figure 3f, much more ROS was generated in the FA–L–CuPP+US group than in the control+US group, which contributed to the good SDT efficiency.

### 2.3. In Vivo SDT Treatment Efficiency of the FA–L–CuPP Nanoparticles

**Figure 4:**
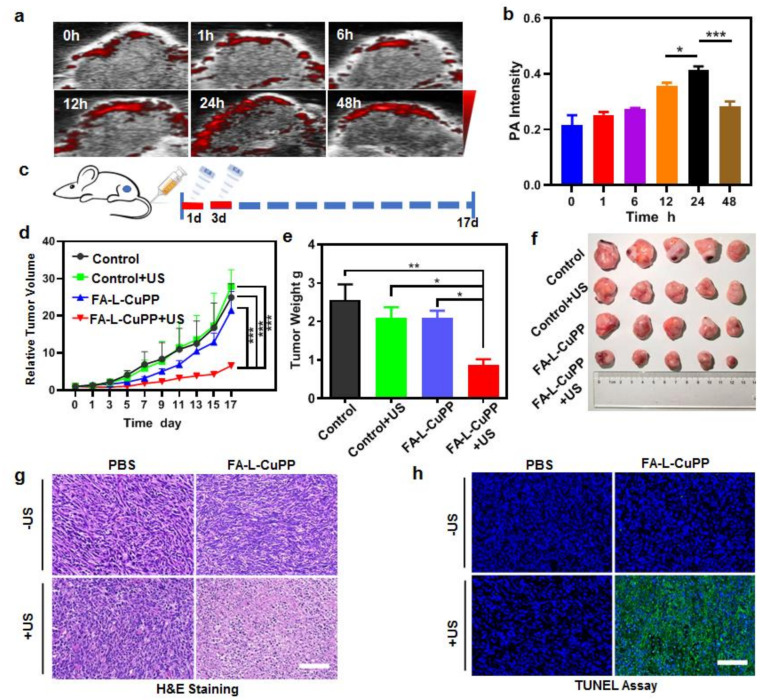
The in vivo accumulation and SDT treatment effect of FA–L–CuPP in mice bearing 4T1 tumors: (**a**) time-dependent in vivo PA imaging after the intravenous injection of the FA–L–CuPP. (**b**) The semi−quantified PA signal intensity of the FA–L–CuPP in tumor (* *p* < 0.05, *** *p* < 0.001). (**c**) In vivo therapeutic protocol of SDT on 4T1 tumor xenograft (1.0 W·cm^−2^, 5 min, 50% duty cycle). (**d**) The tumor growth curves with various treatments (*** *p* < 0.001). (**e**) The excised tumor weights acquired at the end of treatments (* *p* < 0.05, ** *p* < 0.01). (**f**) Photographs of the excised tumors at 17 d after different treatments. (**g**) The tumor sections stained by hematoxylin and eosin (H&E), and (**h**) the TUNEL assay of tumor sections conducted on the different groups (scale bar = 50 µm).

Based on the good SDT effect of FA–L–CuPP against 4T1 cells, we hypothesized FA–L–CuPP would exhibit good in vivo SDT and effectively inhibit tumor growth. To evaluate the optimal therapy time-point, the accumulation of FA–L–CuPP in vivo in a mouse model bearing a 4T1 tumor was assessed. To directly observe the accumulation over time, we used PA imaging to detect the change in photoacoustic (PA) imaging signal in tumor after intravenous injection of FA–L–CuPP. As Figure 4a reveals, the PA signal increased over time, and the maximal accumulation was observed at 24 h, showing the best accumulation in vivo. At 48 h, the PA signal began to recede. The semi-quantified PA signal intensity in Figure 4b also exhibited the highest intensity at 24 h, suggesting the best accumulation, which provided guidance for the effective SDT.

In this study, 4T1 tumor xenograft Balb/c nude mice were used to evaluate the SDT effect by detecting tumor inhibition. The protocol was according to Figure 4c. The US treatment was intervened 24 h after intravenous injection, and the same treatment was conducted again on the third day. As shown in Figure 4d, tumor growth was significantly suppressed in the FA–L–CuPP+US group compared with the other three groups. After US treatment, the US-only and drug-only treatment had no evident efficacy in restricting the growth of tumors, but the FA–L–CuPP+US treatment performed good tumor inhibition, which caused the significant discrepancy in the excised tumor weights in contrast to other groups (Figure 4e). Furthermore, the excised photographic images of the tumor at the end of treatments in Figure 4f show that the FA–L–CuPP+US group had the smallest tumor among these tumors in all the groups, demonstrating the best cure on the mice of the FA–L–CuPP+US group.

Tumor sections at the end of the therapy were carried out by staining hematoxylin and eosin (H&E). Then, the TdT-mediated dUTP nick-end labeling (TUNEL) assay was further conducted to check the tumor cells for necrosis and apoptosis. As revealed in Figure 4g, negligible necrosis was observed in the control group, only US group and drug group, but a small portion of purple-blue (nuclei stained by hematoxylin) area in the FA–L–CuPP+US group was observed, suggesting that the majority of cancer cells were in the state of apoptosis and necrosis. The TUNEL assay results in Figure 4h exhibited that the FA–L–CuPP+US group showed many more apoptotic/necrosis cells than the other control groups, suggesting the inhibition of tumor cell proliferation.

### 2.4. In Vivo Biosafety Evaluation of the FA–L–CuPP Nanoparticles

**Figure 5:**
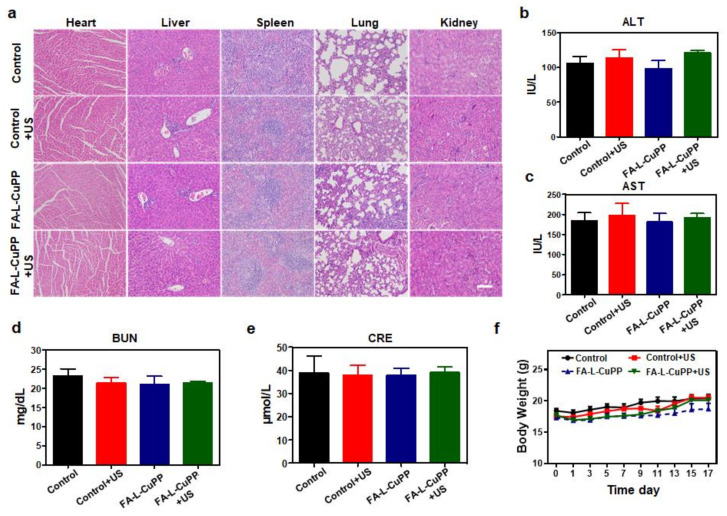
Biosafety evaluation of FA–L–CuPP: (**a**) H&E staining images of the main organs of mice (heart, liver, spleen, lung and kidney, scale bar = 100 μm). (**b**,**c**) ALT/AST (liver function) in serum level was analyzed (n = 5). (**d**,**e**) BUN/CRE (renal function) in serum level was analyzed (n = 5). (**f**) During the 17-day study period, body weights of the mice were recorded under the different conditions.

Biosafety is very important for evaluating the value of sonosensitizers. Therefore, H&E staining of the main organs of mice was first conducted. From the data in Figure 5a, compared with the control, control+US and drug-only group, no obvious adverse effects were observed in various organs in the FA–L–CuPP+US group, suggesting the treatment was biosafe. In addition, at the end of the experiment, ALT/AST and BUN/CRE in serum were carried out to measure liver/renal damage, respectively. From Figure 5b,e, there were a few differences among all groups, implying the insignificant liver and renal toxicity of FA–L–CuPP. Moreover, the changes in mice weight were also monitored to examine biosafety. Figure 5f showed that no obvious variation occurred in mice weight in all the groups during the treatment, indicating the good biosafety of the treatment.

## 3. Conclusions

In summary, we designed a novel urchin-shaped nanosonosensitizer (FA–L–CuPP) through an FA-modified liposome-loading copper-based coordination complex. FA–L–CuPP exhibited a good US-irradiating response and generated large amounts of ROS (such as ^1^O_2_, O_2_^−^ and ⋅OH) under US treatment, likely due to energy conversion from the CuPP complex under US irradiation. Effective SDT was also demonstrated in in vitro 4T1 tumor cells and in vivo 4T1 tumor xenograft Balb/c nude mouse model. The tumors treated by FA–L–CuPP+US irradiation were successfully inhibited with negligible damage on the main organs, suggesting the efficient and noninvasive SDT. Overall, the tumor-targeting nanosystem induced abundant ROS through energy conversion under US energy. This study provided a Cu−based sonosensitizer and explored the possible mechanism, which would provide an idea to design novel sonosensitizers and their application in SDT antitumor and even other therapy modes such as in combination with pharmaceutical agents such as immunostimulatory and chemotherapy agents.

## 4. Experimental Section

### 4.1. The Preparation of FA–L–CuPP Nanoaprticles

To prepare the FA–L–CuPP nanoparticles, a previously reported method was used with slight modifications [25]. Lecithin (50 mg, Avanti Polar Lipids Inc., Alabaster, AL, USA) and cholesterol (17 mg, Aladdin, Shanghai, China) were dissolved in chloroform (CHCl_3_, 10 mL). Then, 1 mL CuPP complex (Bailingwei Technology Co., Ltd., Beijing, China, methyl alcohol solution, 1 mg·mL^−1^), was added to the above mixed solution, followed by 1 mg folate−PEG_2000_−DSPE (Xi’an Ruixi Biological Technology Co., Ltd., Xi’an, China). Then, the solvents were evaporated by decompressed rotary evaporation, and a thin film was formed on the wall of the 50 mL round bottom flask. Vacuum freeze-drying was used to remove the residual solvents in the film. To disperse the dry product, 3 mL ultrapure water was added. After another 5 min ultrasonic treatment in ice-bath, the bright FA–L–CuPP nanoparticles were obtained. After the nanoparticles were purified using a centrifugal filter (cutoff MW: 100 kDa, 4000 rpm), NaHCO_3_ solution (1M) was added to adjust the pH value of 7.4. The nanoparticles without folate (L-CuPP) were obtained using the same method except for the absence of the folate-PEG-DSPE. The morphology was detected by transmission electron microscope (Tecnai G2 F20 S-Twin, Hillsboro, OR, USA). A dynamic light-scattering spectrometer (Zetasizer, Nano ZS, Malvern Instruments, Malvern, UK) was used to analyze the hydrodynamic diameters and zeta potential of nanoparticles. The UV-Vis spectra of nanoparticles were recorded using a UV-Vis spectrometer (Lambda25, Perkin−Elmer, Waltham, MA, USA), and the fluorescence (FL) spectra were carried out by FL spectroscopy (F900, Edinburgh Industries, Livingston, UK).

### 4.2. The Encapsulation Rate Detection

The encapsulation efficiency (EE) was carried out in DMSO using a UV–Vis spectrophotometer according to the absorption of CuPP at 410 nm. The standard curve of CuPP was first conducted in DMSO, and EE was calculated by the following equation: EE = (m_initial_ − m_supernatant_)/m_initial_ × 100%. The m_supernatant_ is the amount of non-encapsulated CuPP in supernatant solution from ultrafiltration tackled with DMSO.

### 4.3. ROS Generation Detection in Solution

The FA–L–CuPP solution (2 mL, CuPP concentration of 40 μg⋅mL^−1^) and singlet oxygen sensor green (SOSG, 100 μg⋅mL^−1^, 5 μL) were evenly mixed in a 35 mm culture dish. SOSG is a popular ^1^O_2_ probe in which FL intensity would increase after reaction with ^1^O_2_. Then, US wave (1.0 W·cm^−2^, 50% duty cycle) was used to irradiate the solution by a US transducer with a columnar US probe (XK-2011R, Wuhan Xingkang, Wuhan, China). ROS FL intensity was measured on a multifunctional microplate reader with λ_ex_ of 488 nm and λ_em_ of 525 nm (Synergy H1 Hybrid, BioTek, Winooski, VT, USA). ESR spectra were obtained by a Bruker EMX electron paramagnetic resonance spectrometer (Bruker EMXnano, Bruker Biospin, Ettlingen, Germany). After TEMPO/DMPO (2,2,6,6-Tetramethylpiperidine/5,5-dimethyl-1-pyrroline-N-oxide, 2.5 μL) was added, ^1^O_2_/·OH signal was then detected in FA–L–CuPP solution (CuPP concentration of 61.5 μmol⋅L^−1^, 2 mL) with or without US treatment.

### 4.4. In Vitro Cytotoxicity of FA–L–CuPP

The cytotoxicity of FA–L–CuPP was tested on the 4T1 cell lines of triple-negative breast cancer with or without US irradiation. Next, 5 × 10^3^ 4T1 cells were seeded into 96-well plates and incubated for a night (37 °C, 5% CO_2_). The cells were then co-incubated with the FA–L–CuPP nanoparticles with different concentrations for 24 h. Then, fresh phosphate-buffered saline (PBS) solution was used to wash the cells three times. Afterwards, the medium was replaced with fresh culture medium with CCK8 (*v*/*v* = 9:1, 100 μL) and was continuously incubated for 1–1.5 h. A microplate reader (λ = 450 nm) was used to detect the absorption. The sonotoxicity was also conducted with a similar experiment to evaluate the cytotoxicity, except that the cells were exposed by a US probe (1.0 W·cm^−2^, 50% duty cycle, 2 min) after incubation for 3 h.

### 4.5. In Vitro Uptake of FA–L–CuPP Nanoparticles

Then, 4T1 cells (1 mL, 1 × 10^5^ cells⋅well^−1^) were seeded in a 12-well plate and incubated for a night (5% CO_2_, 37 °C). Then, the cells were washed twice using PBS and incubated with FA–L–CuPP (10 μg⋅mL^−1^, 1 mL culture medium solution) for different time. The uptake results were obtained by flow cytometry (BD FACSCalibur, Franklin, NJ, USA) after the cells were collected.

### 4.6. ROS Generation Detection In Vitro

Next, 4T1 cells (200 μL, 5 × 10^3^ cells⋅well^−1^) were incubated in 8-well chambered cover glasses for a night. Then, 200 μL FA–L–CuPP culture medium solution (10 μg⋅mL^−1^) replaced the old culture medium and incubated for 3 h. DCFH-DA (1 mg⋅mL^−1^ DMSO, 1 μL) was added. Then, 30 min later, the cells were washed twice with PBS and tackled with US treatment (1.0 W·cm^−2^, 50% duty cycle, 2 min). After a 30 min incubation, the cells were fixed with paraformaldehyde solution (100 μL, 4%, 20 min) and stained with DAPI (100 μL, 1 μg/mL, 10 min, Invitrogen, Carlsbad, CA, USA). Confocal laser scanning microscopy imaging (CLSM, TCS SP5II, Leica, Weztlar, Germany) was used to explore ROS FL signal. ROS FL intensity was then detected using flow cytometry (BD FACSCalibur, Franklin, NJ, USA) with the similar method above, except the 8-well chambered cover glasses were replaced by 12-well plates (1 × 10^5^ cells⋅well^−1^, 1 mL).

### 4.7. Animals

Six-week-old healthy female Balb/c nude mice were obtained from Vital River Animal Technology Co. Ltd (Tongxiang, Zhejiang, China). and were used for in vivo evaluation. Protocols were approved by the Animal Care and Use Committee (Shenzhen Institutes of Advanced Technology, Chinese Academy of Sciences, Shenzhen, China).

### 4.8. The In Vivo Accumulation Evaluation of FA–L–CuPP Nanoparticles

Firstly, 4T1 tumor cells were inoculated on the BALB/c nude mice and raised for several days. When the tumor volume was approximately 200 mm^3^, FA–L–CuPP (500 μg⋅mL^−1^, 200 μL) was intravenously injected into the mice. Then, the PA signal in vivo was recorded by a preclinical photoacoustic computerized tomography scanner (Vevo LAZR-X, Toronto, ON, Canada) at different time points.

### 4.9. In Vivo SDT Efficacy and Mechanism Measurement

To evaluate the SDT effect in vivo, Balb/c nude mice with 4T1 tumors were constructed by subcutaneously implanting 4T1 cells in medium (100 µL, 1 × 10^6^) into the right thigh of mice. After the tumor volume reached approximately 100 mm^3^, the tumor-bearing mice were randomly divided into four groups (only PBS, PBS+US, only FA–L–CuPP and FA–L–CuPP+US, n = 5 in each group). The FA–L–CuPP nanoparticles (200 μL, 500 μg⋅mL^−1^) were intravenously injected in the FA–L–CuPP group, and the FA–L–CuPP+US group. Then, 24 h later, the mice were treated with or without ultrasound irradiation (1.0 W·cm^−2^, 5 min, 50% duty cycle). The same intervention was conducted for the second time on the third day. For the therapy effect detection, the tumor volumes were recorded every 2 days, based on the protocol (V = (ab^2^)/2, a is the length of the tumor and b is the width, respectively). After 17 days, the mice were killed, and the tumors were dissected and weighed. The collected tumors and major organs (heart, lung, liver, spleen, and kidneys) were cut into 8 μm sections. Then, the sections were used for histological analysis by hematoxylin and eosin (H&E) staining or TdT-mediated dUTP nick-end labelling (TUNEL).

### 4.10. Statistical Analysis

All the quantitative values are expressed as mean ± SD. One-way ANOVA analysis followed by Tukey’s post-test was used to assess statistical analysis. The asterisk sign was considered statistically significant.

### 4.11. Calculation Method

HOMO/LUMO orbitals of CuPP complex calculated by DFT at the B3LYP Hybrid function, the 6-311G* (C, N, H, O) and def2tzvp (Cu) basis level.

## Data Availability

The data presented in this study are available on request from the corresponding author upon reasonable request.
